# Local Adaptation With Gene Flow Has a Polygenic Basis in a Threatened Coastal Songbird

**DOI:** 10.1002/ece3.74506

**Published:** 2026-10-18

**Authors:** Jonathan D. Clark, Haw Chuan Lim, Brian J. Olsen, Adrienne I. Kovach

**Affiliations:** ^1^ Department of Natural Resources and the Environment University of New Hampshire Durham New Hampshire USA; ^2^ Fuller Evolutionary Biology Program, Cornell Lab of Ornithology Cornell University Ithaca New York USA; ^3^ Department of Biology George Mason University Fairfax Virginia USA; ^4^ Center for Conservation Genomics National Zoo and Conservation Biology Institute Washington DC USA; ^5^ School of Biology and Ecology The University of Maine Orono Maine USA

## Abstract

Species that span heterogenous environments often exhibit local adaptation despite gene flow among populations. In such cases, the maintenance of local adaptation is controlled by several factors, including the strength of selection, migration, assortative mating, and genomic architecture. The song sparrow ( *Melospiza melodia* ) is a widespread songbird with diverse subspecies, including the coastally adapted Atlantic song sparrow (*M. m. atlantica*). Despite extensive gene flow with a generalist subspecies, Atlantic song sparrow populations have been found to maintain their coastally adaptive phenotypes. We produced a new chromosome‐level reference assembly for the species and whole‐genome data to explore the genomic architecture of divergence with gene flow and the genetic basis of adaptation in this subspecies. Adaptation in the Atlantic song sparrow has a polygenic basis, with loci under divergent selection scattered throughout the genome rather than concentrated in low‐recombination linkage blocks. Signatures of fine‐scale population structure, limited genomic differentiation, and divergent selection on a small set of genes suggest a role for natural selection in maintaining this subspecies boundary. Our findings highlight the complex interplay among selection, gene flow, and recombination in shaping adaptation with gene flow and provide insights into the genomic architecture of ecological divergence within a widespread, morphologically diverse species.

## Graphical abstract

Using a new chromosome‐level reference genome and whole‐genome sequencing, we show that the threatened, coastally specialized Atlantic song sparrow ( *Melospiza melodia atlantica* ) maintains adaptive divergence from a widespread generalist subspecies with divergently selected loci scattered across the genome rather than concentrated in low‐recombination linkage blocks. These findings, together with signatures of fine‐scale population structure and limited genome‐wide differentiation, point to selection as the primary force maintaining this subspecies boundary despite extensive gene flow.

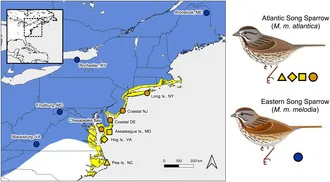

## 1 Introduction

Species with broad ranges that span heterogeneous environments often exhibit heritable trait divergence among populations due to differing selection pressures, i.e., local adaptation (Rundle and Nosil 2005; Savolainen et al. 2013). Locally adapted populations may experience maladaptive gene flow where the ranges of adjacent, divergently adapted populations abut (Lenormand 2002). When divergently adapted subpopulations experience extensive gene flow, differentiation typically becomes limited to the genomic regions that underpin local adaptation while the neutral genomic background is homogenized among populations (Feder et al. 2012; Tigano and Friesen 2016). Consequently, high‐resolution genomic data are necessary to detect population structure in such cases (Funk et al. 2012). However, such cases also present an opportunity to identify the genetic basis of local adaptation, as loci under divergent selection should be easily detectable against the low differentiation of the genomic background (Tigano and Friesen 2016).

Systems where local adaptation is maintained despite gene flow can also provide insights into the early processes of ecological speciation, as ecologically driven divergence among interbreeding populations can eventually lead to ecological speciation under certain circumstances. For example, ecological speciation can occur between divergently adapted populations that interbreed when selection is sufficiently strong to create post‐zygotic reproductive isolation (Rundle and Nosil 2005). Ecological speciation can also result when assortative mating becomes coupled with ecologically driven trait divergence, creating pre‐zygotic barriers, as in the case of so‐called “magic” traits (Servedio et al. 2011).

The ability of interbreeding populations to maintain adaptive divergence despite gene flow is controlled by several factors, including the strength of selection, extent of migration, effects of assortative mating, and interactions between physical linkage within the genome and genomic architecture (Tigano and Friesen 2016). In the simplest cases, where a single causal locus of large effect underpins a locally adaptive phenotype, strong selection alone may be sufficient to maintain divergence among interbreeding populations (e.g., von Holdt et al. 2018; Barrett et al. 2019; Miranda et al. 2021). However, when the genomic architecture of local adaptation involves the effects of numerous genes, it is common for divergent loci to be restricted to recombination cold spots in the genome—such as centromeres and inversions—causing sets of co‐adaptive alleles to avoid recombination and be inherited as a linkage block (Burri et al. 2015; Ravinet et al. 2017).

There are several examples of adaptation to different selective regimes maintained by chromosomal inversions in diverse species, including mice (Hager et al. 2022), birds (Taylor and Campagna 2016; Funk et al. 2021), marine fishes (Berg et al. 2017; Wilder et al. 2020; Akopyan et al. 2022), and snails (Koch et al. 2021). In three closely related, hybridizing bumble bee species (genus *Bombus*), loci resistant to gene flow among species are limited to genomic regions around centromeres (Christmas et al. 2021). These cases illustrate that for populations with extensive gene flow to maintain divergent adaptations typically require either strong selection with a simple genetic basis or that co‐adaptive gene complexes under divergent selection be co‐located in regions of suppressed recombination.

On the other hand, theory predicts that non‐independence of beneficial and deleterious alleles throughout the genome due to linkage prevents selection from acting efficiently (Hill and Robertson 1966). Some experimental evidence has supported this, demonstrating that a higher recombination rate can more effectively incorporate new beneficial mutations while eliminating deleterious ones (Haddrill et al. 2007). Therefore, when the genetic basis of adaptation is polygenic, high recombination could result in the more efficient sorting of beneficial sets of alleles in response to strong selection.

A species that provides an ideal opportunity to study the basis of local adaptation with gene flow is the song sparrow ( *Melospiza melodia* ), a widespread North American songbird that exhibits phenotypic differentiation related to its regional environment. The song sparrow is a complex of 25 recognized subspecies, each associated with a specific breeding habitat ranging from subarctic islands to hot southwestern deserts (Aldrich 1984; Patten and Pruett 2009). The overlap between subspecies' ranges and climate zones suggests that the phenotypic diversity of this species is largely a result of local adaptation across its range (Aldrich 1984; Patten and Pruett 2009). The ranges of several subspecies abut in broad contact zones, where there is the opportunity for gene flow. Thus, for these parapatric subspecies pairs to remain phenotypically divergent in the absence of intrinsic genetic incompatibilities requires mechanisms of assortative mating, divergent natural selection, or some combination of the two.

Some song sparrow subspecies are widespread generalists, while other subspecies are geographically and ecologically restricted specialists. The Atlantic song sparrow (*M. m. atlantica*) is a specialist subspecies of the song sparrow that is associated with tidal salt marsh and coastal dune habitats in eastern North America, found breeding from the Outer Banks of North Carolina to Cape Cod, Massachusetts (Figure 1; Todd 1924; Patten and Pruett 2009). Many avian species specialized to tidal salt marshes and coastal dunes are experiencing precipitous population declines due to coastal degradation and sea level rise (Correll et al. 2017; Field et al. 2017). Some data suggest the Atlantic song sparrow has declined by as much as 97% in portions of its range over the last three decades (Watts 2025).

**FIGURE 1:**
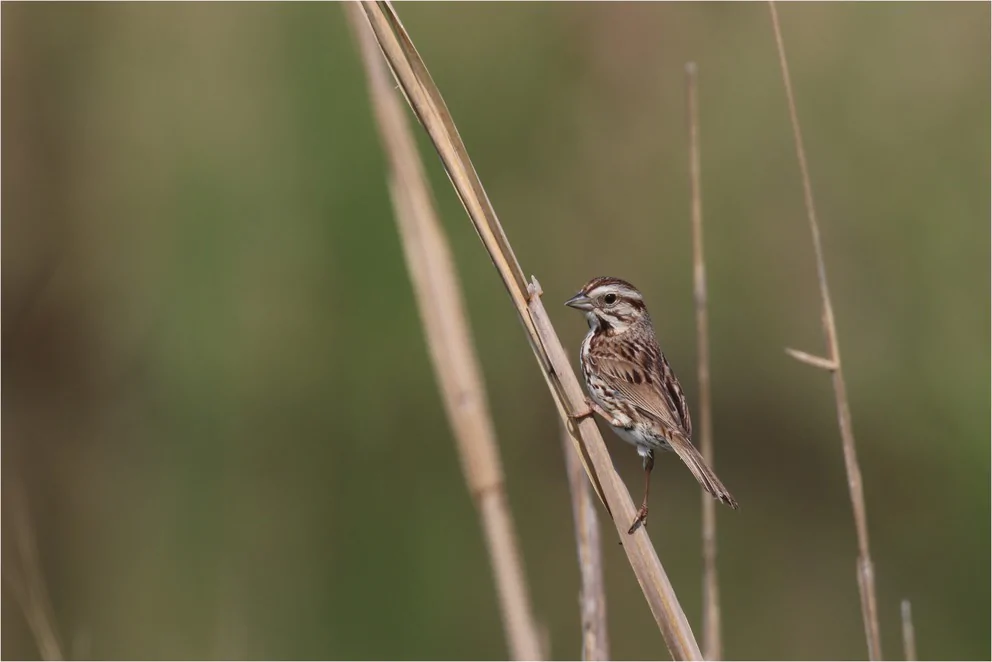
Photo of an Atlantic song sparrow during the breeding season in the marshes of the Chesapeake Bay. Taken by Gabriel Foley (Macaulay Library 583942411).

Coastal habitats present many evolutionary challenges to terrestrial vertebrates, such as high salinity, limited access to freshwater, high heat with limited cover, and periodic tidal flooding. The Atlantic song sparrow exhibits adaptive phenotypes typical of other avian species adapted to tidal habitats (known as the “salt marsh syndrome”; Greenberg et al. 2006), including a large bill, plumage with more pronounced dark streaking, and, implicitly, the ability to tolerate or avoid high salt intake (Figure 2). Each of these three divergent phenotypes has hypothesized adaptive significance.

**FIGURE 2:**
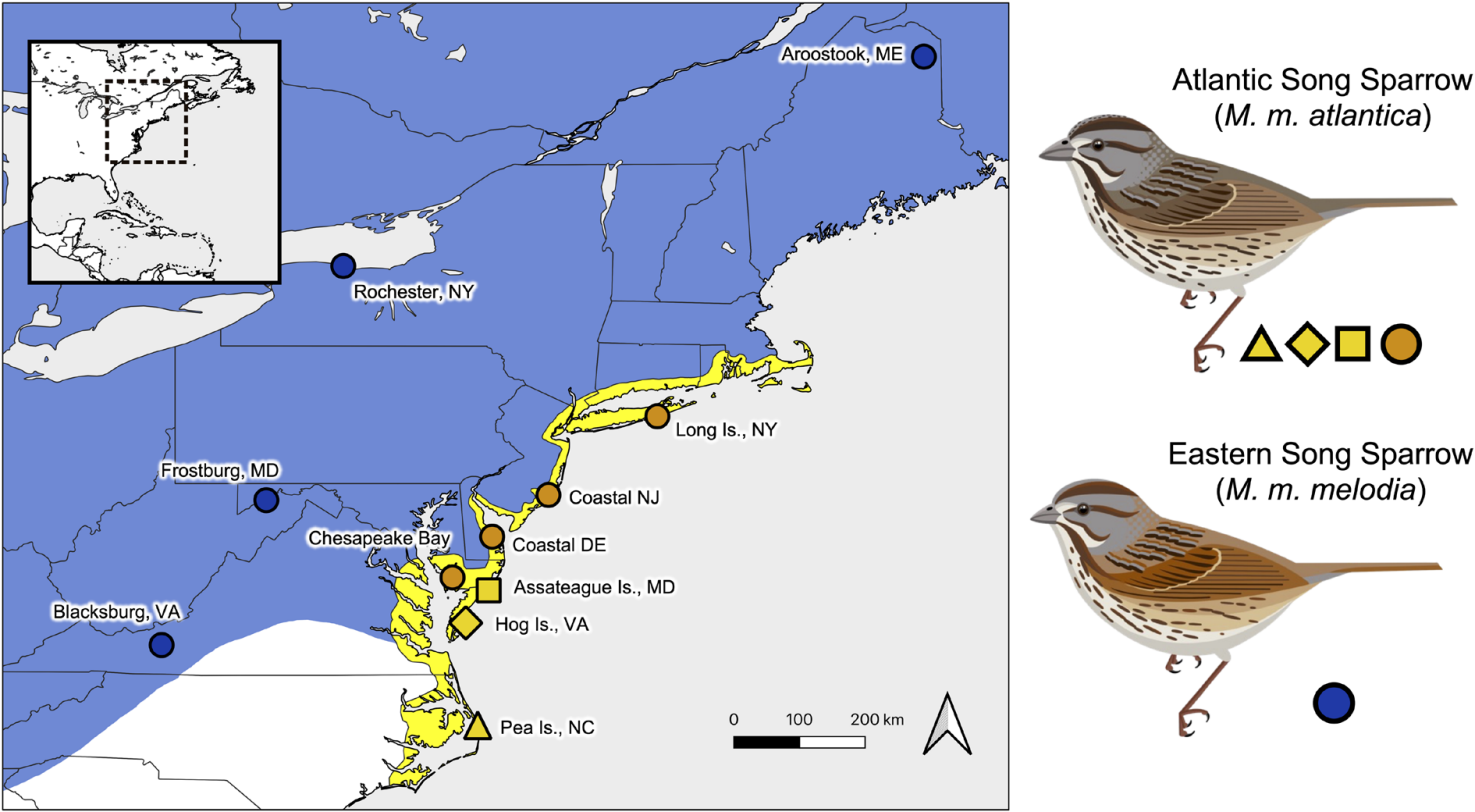
Map of sampling sites. *Left*, Range of the Atlantic song sparrow (yellow area) and eastern song sparrow (blue area) in the northeastern United States. Points represent sampling locations and are color‐coded by population grouping, where blue represents inland sites, orange represents coastal mainland sites, and yellow represents southern barrier islands. Inset map of eastern North America showing study region in the dashed box. *Right*, Illustrations of each subspecies by Alex Lehner. The Atlantic song sparrow is distinguished from the eastern song sparrow by its larger bill, grayer plumage, and darker/heavier streaking.

The adaptive basis of a large bill in these habitats serves to reduce evaporative water loss during thermoregulation via two primary functions. First, the higher surface‐area of a larger bill functions to increase non‐evaporative cooling as blood can be shunted to the bill for radiative cooling—that is, the Greenberg‐Tattersall Corollary of Allen's Rule (Greenberg et al. 2012; Danner and Greenberg 2015; Olsen et al. 2019). Second, increased bill volume provides greater space for internal vascular surface area in the nasal turbinates which likely reduces water loss during respiration (Danner et al. 2016). Darker plumage has been suggested to benefit bird species in coastal environments, as increased eumelanin (black pigment) content prevents microbial degradation of feathers by keratinolytic bacteria, which are more abundant and potent in hot, moist habitats (Goldstein et al. 2004; Gunderson et al. 2008; Peele et al. 2009). Some passerine species adapted to saline environments exhibit physiological adaptations to cope with salt absorption and excretion (Goldstein 2006). Though no studies have thoroughly assessed salt intake or excretion ability in the Atlantic song sparrow, the subspecies is capable of excreting hyperosmotic urine (Clark 2024) and known to consume osmoconforming prey (Rumfelt 2024).

In the Atlantic song sparrow, these potentially adaptive phenotypic differences exist despite adequate opportunity for gene flow with morphologically distinct inland populations. Specifically, throughout the majority of the Atlantic song sparrow range, it is parapatric with the eastern song sparrow (*M. m. melodia*), a widespread generalist subspecies found breeding in a variety of shrubby habitats east of the Rocky Mountains (Patten and Pruett 2009). Notably, the eastern song sparrow breeds in coastal habitats north of the Atlantic song sparrow range, demonstrating its ability to utilize these habitats at higher latitudes where summer temperatures are cooler.

Despite high levels of gene flow detected between these subspecies across their broad contact zone, populations of the Atlantic song sparrow appear to maintain their coastally adaptive phenotypes (Clark et al. 2022). Phenotypic differences between the Atlantic and eastern song sparrows, however, are subtle and, at sites proximal to the coast, clinal (Patten and Pruett 2009; Clark et al. 2022), suggesting that the genetic basis of local adaptation in the Atlantic song sparrow is likely polygenic. Therefore, causal genes underpinning adaptive divergence may be localized to regions of suppressed recombination or, alternatively, these genes may be randomly scattered throughout the genome if selection is sufficiently strong and recombination is high.

Here, we examined the genome of the Atlantic song sparrow across its range in comparison to the eastern song sparrow subspecies to explore the genomic architecture of divergence with gene flow and uncover the genetic basis of coastal adaptation in this subspecies. We produced a new chromosome‐level reference assembly for the song sparrow and used whole‐genome resequencing of individuals across the ranges of the Atlantic and eastern song sparrow subspecies to (1) characterize population structure, (2) discern the genomic landscape of divergence with gene flow in this system, and (3) identify candidate genes for coastal adaptation in the Atlantic song sparrow. We hypothesized that genes under selection were likely to be associated with traits of the salt marsh syndrome, such as osmoregulation (salinity tolerance), skeletal development (bill size), or melanin synthesis (plumage pigmentation).

## 2 Materials and Methods

### 2.1 Sample Collection

We sampled song sparrows during the breeding season (May–August) across the eastern United States in 2021 and 2022, choosing sites representative of the entire Atlantic song sparrow range and an equal breadth of the eastern song sparrow range in the states of Maine (ME), New York (NY), New Jersey (NJ), Delaware (DE), Maryland (MD), Virginia (VA), and North Carolina (NC). In total, we sampled 11 sites, targeting ≥ 5 individuals per site (Figure 2); Table S1. Sites were assigned to one of three categories. Inland sites in the eastern song sparrow range were categorized as “inland” and were specifically chosen to be > 250 km from the Atlantic song sparrow range to prevent the potential for any admixture (Clark et al. 2022). Coastal sites in the Atlantic song sparrow range were divided into two categories. The southernmost sites on barrier islands (Assateague, Hog, and Pea islands), where there was little‐to‐no chance of direct gene flow with inland birds, were categorized as “barrier islands.” The remaining coastal sites on the mainland—where there was the possibility of admixture with birds from nearby inland habitats—were categorized as “coastal mainland.”

We used mist‐nets and audio playback to capture adult song sparrows, then to confirm that each bird was a member of the targeted breeding population, we identified breeding condition by the presence of a cloacal protuberance (breeding male) or a brood patch (breeding female). A blood sample (50–100 μL) was collected from each bird via the brachial vein and stored in 500 μL Queen's lysis buffer at room temperature. Birds were banded with a uniquely numbered aluminum band issued by the United States Geological Survey before release. Sampling methods were approved by the University of New Hampshire Institutional Animal Care and Use Committee (approvals 190401 and 220401) and conducted with proper federal, state, and local banding permits. Additional blood samples in Queen's lysis buffer from Rochester, NY were provided by the Braddock Bay Bird Observatory, and some samples were taken from a prior study (from Assateague, MD, Blacksburg, VA, Claytor Lake, VA, and Frostburg, MD; Clark et al. 2022).

In total, blood samples were collected from 60 individuals. DNA was extracted from each blood sample using the Quick‐DNA Miniprep Kit (Zymo Research) following manufacturer specifications with 50 μL of blood‐lysis solution and a one‐hour digestion. DNA was eluted using 200 μL of 10 mM Tris–HCl. The concentration of double‐stranded DNA in each extract was quantified using a Qubit 4 Fluorometer (ThermoFisher Scientific) with the high‐sensitivity assay kit.

### 2.2 Reference Genome Assembly

We generated a new, high‐quality reference genome for the song sparrow in collaboration with the Vertebrate Genomes Project (VGP; Rhie et al. 2021) from a single additional individual. For the reference individual, we selected a breeding‐condition female song sparrow (band 2831‐61162) of the nominate subspecies that was captured in the salt marsh edge at Wharton Point on Maquoit Bay in Brunswick, ME (Coordinates: 43.869° N, 69.993° W) on July 29th, 2020. We collected 100 μL of blood in 100% ethanol before releasing the bird and stored the sample at −20°C.

DNA was extracted from this sample by the VGP and sequenced at 30.5 X coverage on a Sequel II HiFi (PacBio), producing long‐read data. Additionally, Hi‐C data were generated using the Arima Genomics Hi‐C kit 2 at 103.7 X coverage. The standard VGP assembly pipeline (v. 2.0) was used to generate a genome assembly from these data, which included *hifiasm* (v. 0.18.8), *solve* (v. 3.7), and *YaHS* (v. 1.2a.2). Raw data, sequence evaluations, and curated assemblies can be found on the VGP Genome Ark page for the song sparrow (https://www.genomeark.org/genomeark‐all/Melospiza_melodia.html). The National Center for Biotechnology Information (NCBI) Eukaryotic Genome Annotation Pipeline was used to annotate the resulting reference assembly. The final reference assembly was submitted to the NCBI Reference Sequence Database. Syteny with the white‐throated sparrow ( *Zonotrichia albicollis* ) genome (GCF_047830755.1) was assessed using *RagTag* (v. 2.0.01; Alonge et al. 2022).

### 2.3 Whole‐Genome Re‐Sequencing

Sequencing libraries were generated for each individual using the KAPA HyperPlus Library Prep Kit (Roche) following manufacturer protocol with 250 ng of genomic DNA suspended in a 35 μL volume. To conserve supplies, half volumes of kit reagents were used, diluted to the correct volume with 10 mM Tris–HCl, for each step of the library preparation. The KAPA Unique Dual‐Indexed Adapter Kit (Roche) was used to barcode library molecules with a unique dual‐index to minimize the possibility of index hopping (MacConaill et al. 2018). Following enzymatic fragmentation (digested at 37°C for 20 min) and end repair and A‐tailing (ERAT; at 65°C for 30 min), samples were quantified and equimass pooled in groups of 6 for size selection (*n* = 10); samples were randomized across pools to prevent the confounding of population structure with potential batch effects. Size selection of 500–850 bp library molecules was performed for each pool using a lane of a BluePippin (Sage Science) with a 1.5% agarose gel cassette.

Low‐cycle PCR was used to amplify post‐size‐selection library pools (3 cycles) with KAPA HiFi HotStart ReadyMix (Roche) and Illumina TruSeq Primer Mix (Roche). After an initial denaturation at 98°C for 45 s, PCR cycle conditions followed manufacturer guidelines: denaturation at 98°C for 15 s, annealing at 60° for 30 s, extension at 72° for 30 s. After the final PCR cycle, there was a final extension at 72°C for 1 min, and the PCR products were then held at 4°C.

Post‐PCR library pools were purified using SPRI beads (Beckman Coulter) and quantified. Post‐PCR library pools were then assessed using a TapeStation (Agilent) and equimolar pooled into two final libraries (containing 30 samples each) for submission. The two final libraries were submitted to the Genomic Sciences Laboratory at North Carolina State University for sequencing on one lane each of a NovaSeq 6000 (Illumina) with S4 chemistry (150 bp, paired‐end reads) and 5% PhiX spike‐in, targeting approximately 15X coverage.

### 2.4 Variant Discovery and SNP Filtering

Sequencing data were received demultiplexed by unique, dual‐index barcodes, and *FastQC* (v. 0.11.5) was used to assess the quality of each library. The program *AdapterRemoval* (v. 2.3.1) was then used to trim and merge reads, remove adapter sequences, and filter for Phred quality (‐‐trimns, ‐‐trimqualities, ‐‐minquality 35, ‐‐minlength 25; Schubert et al. 2016). Reads were aligned to the reference genome using *Bowtie 2* (v. 2.4.1; ‐‐phred33, ‐‐very‐sensitive‐local, ‐I 149, ‐X 900), and the resulting BAM files were sorted using *Samtools* (v. 1.10; Li et al. 2009; Langmead and Salzberg 2012). Next, *Picard* (v. 2.18.1) was used to validate, mark duplicate reads in, and index the resulting BAM files. The program *Qualimap* (v. 2.2.1) was used to examine the filtered alignments (García‐Alcalde et al. 2012).

The *mpileup* and *call* commands of the program *BCFTools* (v. 1.10.2) were used to call genotypes from the alignments (‐d 1000, ‐‐min‐MQ 30) and genotypes were output as a VCF file using the multiallelic caller (Li et al. 2009). Next, *VCFTools* (v. 0.1.16) was used to filter the resulting raw VCF to only retain biallelic single nucleotide polymorphisms (SNPs; ‐‐min‐alleles 2, ‐‐max‐alleles 2, ‐‐remove‐indels) with less than 20% missing data (‐‐max‐missing 0.8), an average depth of > 8 X and < 50 X (‐‐min‐meanDP 8, ‐‐max‐meanDP 50), and an average site quality > 30 (‐‐minQ 30; Danecek et al. 2011). A minor allele frequency (MAF) > 5% (‐‐maf 0.05) was then applied. From the resulting VCF, *VCFTools* was used to assess the average depth of coverage for each individual (‐‐depth) and pairwise relatedness (‐‐relatedness2). Individuals with an average depth < 7 X (*n* = 5) and an individual from each pair of close relatives (*n* = 2)—defined here as 3rd‐degree relatives or closer (Phi > 0.06; Manichaikul et al. 2010)—were removed from the dataset using *VCFTools* (‐‐remove), retaining 53 total individuals.

When removing individuals to generate the final filtered dataset, the above *VCFTools* filters were reapplied to the dataset and individual genotypes with a depth < 5 X were converted to missing data (‐‐minDP 5). Finally, individuals in this MAF‐filtered dataset were assessed for the proportion of missing SNPs per individual using *VCFTools* (‐‐missing‐indv), which was < 20% for all individuals. For analyses based on the site frequency spectrum that require no MAF filter and the inclusion of invariant positions, the removed individuals were also excluded from the raw VCF produced by *mpileup* and *call* without any additional SNP filtering.

### 2.5 Linkage Pruning and Statistical Phasing

Analyses of population structure and selection scans (see below), required additional SNP filtering of the MAF‐filtered dataset: SNPs were pruned for linkage disequilibrium (LD) for population structure analyses, and SNPs were converted to statistically phased haplotypes for selection analyses. First, to examine the degree of LD in these data, linkage decay (using mean *r* ^2^) was calculated with the program *PopLDdecay* (v. 3.42) at a maximum window size of 300 kbp (‐MaxDist 300; Zhang et al. 2019) using the MAF‐filtered dataset. Then, an LD‐pruned dataset was created with *BCFTools* (+prune) by excluding one from each pair of SNPs in the MAF‐filtered dataset within a window of 25 kbp (‐w 25000) that had an *r* ^2^ > 0.10 (‐l 0.1). To create a statistically phased haplotype dataset, the program *SHAPEIT4* (v. 4.2.2) was run on the MAF‐filtered dataset (that included all 53 individuals) with a window size of 500 kbp on each scaffold (Delaneau et al. 2013). Only the 62/353 scaffolds that were > 1 Mbp were included in the phasing, however, 9 of these scaffolds contained no SNPs that passed filters and were, therefore, dropped. Thus, the phased haplotype dataset represented 96.4% of the total genome. VCFs of phased scaffolds were then combined into a single phased VCF with *BCFTools* (concat). Finally, *VCFTools* (‐‐keep) was used to subset the phased VCF into individual VCFs for each of the three subpopulations: barrier islands (*n* = 20), coastal mainland (*n* = 15), and inland (*n* = 18).

### 2.6 Population Structure

To explore population structure, a principal components analysis (PCA) was conducted on the LD‐pruned dataset. The PCA was executed in *R* using the *dudi.pca* function of the *ade4* (v. 1.7–22) package without scaling (Dray and Dufour 2007). Missing genotypes were imputed as the mean genotype across individuals, where genotypes were scored as 0, 1, or 2. Then, to test the ability of PCA to reliably discriminate a priori populations, a discriminant analysis of principal components (DAPC) was run using the *adegenet* package (v. 2.1.10; Jombart 2008; Jombart et al. 2010). Three DAPCs were conducted: (a) with subspecies assignment as group (*melodia* or *atlantica*; *K* = 2), (b) with assigned population as group (inland, coastal mainland, or barrier island; *K* = 3), and (c) with assigned population as group but each barrier island separated as its own population (*K* = 5). All DAPCs were performed retaining the first 4 PCs (9.9% cumulative variance), as subsequent PCs were generally uninformative of population structure.

### 2.7 Genome‐Wide Divergence

Using pixy (v. 1.2.10.beta2), fixation index (*F*_ST_; Weir and Cockerham 1984), nucleotide diversity (*π*), and absolute sequence divergence (*d*_xy_) were calculated across the genome in non‐overlapping, 10‐kbp windows to characterize the genomic landscape of divergence between subspecies from the VCF that included invariant positions and did not have a MAF filter (Korunes and Samuk 2021). Because of the possibility for gene flow between the eastern and Atlantic subspecies at coastal mainland sites, these statistics were calculated for the most divergent groups based on PCA, inland birds (*n* = 18) and barrier island birds (*n* = 20).

### 2.8 Selection Scan

When selection favors a particular allele, linkage disequilibrium can result in decreased haplotype diversity in regions of the genome flanking that allele. Therefore, genomic regions that exhibit decreased haplotype diversity around a specific allele in a particular population can indicate local selection acting on that region of the genome (Sabeti et al. 2007). These signatures of selection can be summarized with the cross‐population extended haplotype homozygosity statistic (xpEHH), which is elevated when a particular haplotype is strongly favored in one population while remaining polymorphic in another (Sabeti et al. 2007). The *R* package *rehh* (v. 3.2.2) was used to conduct a haplotype‐based selection scan on the phased data (Gautier et al. 2017) based on xpEHH to identify specific loci exhibiting signatures of divergent selection between the Atlantic and eastern song sparrow subspecies. The selection scan was conducted by independently comparing the two coastal groups (barrier islands and coastal mainland) to the inland population.

First, the statistically phased VCFs of each group (inland, coastal mainland, and barrier islands) were read into *R* for each chromosome using the function *data2haplohh* without polarization. The data were then filtered to remove alleles with low minor allele frequency (< 5%) within each group using the *subset* function. Next, the integrated extended haplotype homozygosity (iES) statistic was calculated across each chromosome for each population using the *scan_hh* function. The integrated haplotype homozygosity statistic (iHS) was then calculated for each population from iES using the function *ihh2ihs*. Finally, the xpEHH statistic was calculated between each subpopulation pair using the *ies2xpehh* function (Sabeti et al. 2007). The two‐sided *p*‐values of xpEHH scores with a false discovery rate (FDR) correction (p.adjust.method = “fdr”) were used to identify markers in regions exhibiting signatures of divergent selection between these subspecies, where a marker with ‐log(*p*‐value) > 10 was considered significant.

### 2.9 Identifying Genes Associated With Coastal Adaptation

Genes were identified around markers exhibiting signals of selection based on xpEHH and considered as putative candidates of divergent adaptation between the subspecies. Gene symbols in the reference annotation (GCF_035770615.1) that overlapped or occurred within 10 kbp of each outlier marker were retrieved. Genes identified as under selection from both the inland–barrier islands and inland–coastal mainland comparisons were considered to have the strongest support for association with coastal adaptation in this system. The program *PANTHER* (v. 18.0), the UniProt database, and the literature were then queried for information on the putative functions of these identified candidate genes (Thomas et al. 2022; The UniProt Consortium 2023).

## 3 Results

### 3.1 Reference Genome

The final reference assembly (bMelMel2.pri.cur.20230821; GCF_035770615.1) was assembled into a total of 353 scaffolds, including 31 complete autosomal chromosomes (some with unlocated scaffolds) as well as the complete Z and W chromosomes. The three smallest micro‐chromosomes were not identified. The contig N50 was 23.12 Mbp and the scaffold N50 was nearly 3.6 times as large at 82.36 Mbp. In total, the assembly contained 20,371 annotated genes. At 1.55 Gbp, the final assembly was larger and more contiguous than recent song sparrow genome assemblies for other subspecies—*M. m. maxima*, *M. m. morphna*, and *M. m. gouldii*—which range from 0.98–1.40 Gbp (Feng et al. 2020; Louha et al. 2020; Benham, Cicero, et al. 2024; GCA_036924335.1). The wide discrepancy in recent reference assembly size among song sparrow subspecies merits further attention to determine whether it is solely the result of technical artifacts of genome assembly methodologies or a biological phenomenon (Benham, Cicero, et al. 2024).

### 3.2 Genomic Data

The final VCF of re‐sequenced birds contained 20 individuals from southern barrier islands, 15 from the coastal mainland, and 18 from inland sites. After all filtering, average individual coverage was 14.6 X (ranging from 7.0–31.6 X), and average genotype missingness was 2.5% (ranging from 0.03%–19.5%). Before MAF and individual genotype depth filtering, the dataset contained 37,255,533 biallelic SNPs, of which 11,496,474 SNPs were retained in the final MAF‐filtered dataset. The phased haplotype dataset retained the majority of SNPs from the MAF‐filtered dataset, 11,493,090. Linkage disequilibrium was notably low in these data, as linkage decayed to a genome‐wide average of *r* ^*2*^ < 0.10 at a distance of 310 bp, decreasing to *r* ^*2*^ = 0.03 at a distance of 300 kbp (Figure A1). A total of 1,114,777 SNPs were retained in the LD‐pruned dataset.

### 3.3 Population Structure

PCA clearly delineated individuals by population grouping in the first two principal components, with PC1 (3.4% variance explained) segregating subspecies (Figure 3). All birds from inland sites were separated from coastal birds and tightly clustered together, with birds from southern barrier islands separating to the other extreme and coastal mainland sites falling between. PC2 (2.4%) primarily discriminated birds from Pea Island, North Carolina from the other two southern barrier island populations. Birds (*n* = 4) from the eastern shore of the Chesapeake Bay (from below 38.5° N) and the Atlantic coast of Delaware (from below 38.7° N) clustered slightly more with Assateague Island birds than with other coastal mainland sites.

**FIGURE 3:**
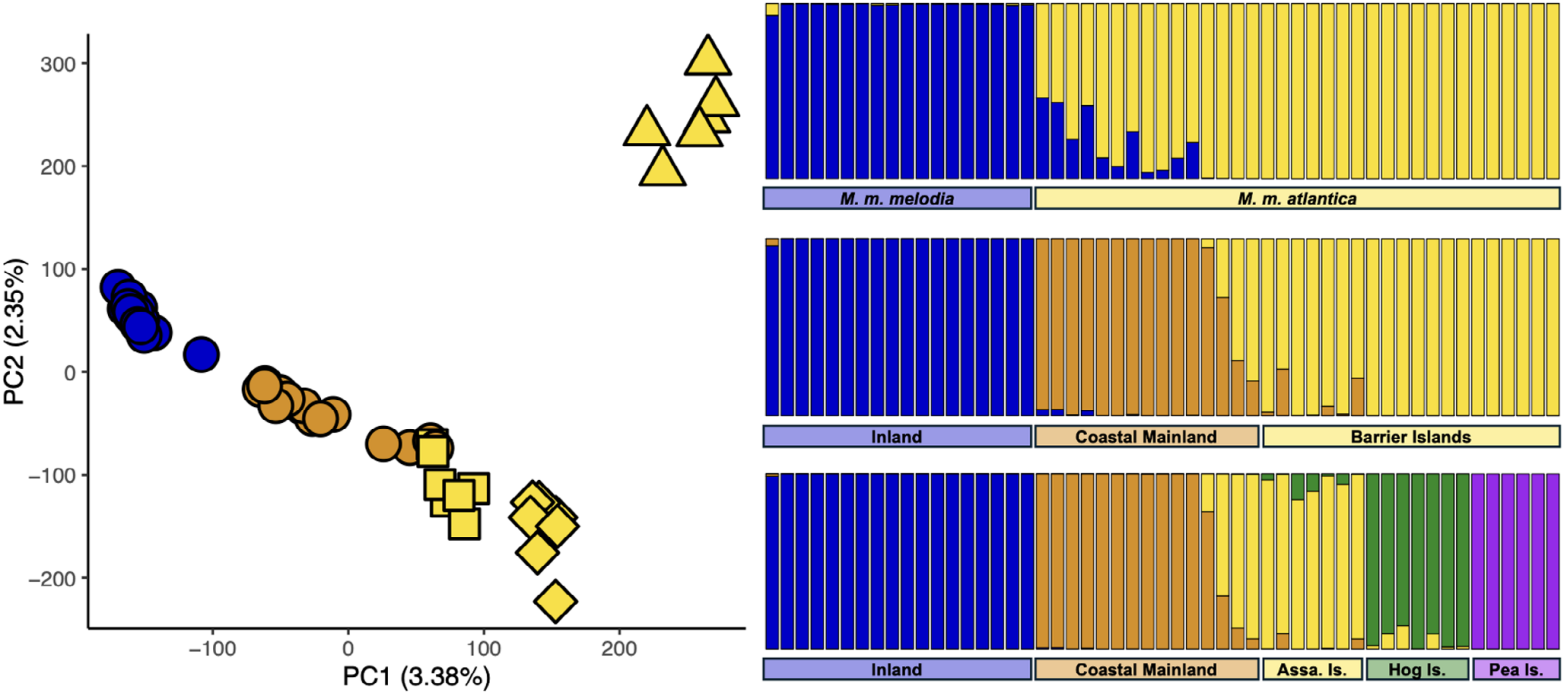
Population structure of the Atlantic and eastern song sparrow subspecies. *Left*, Principal components analysis (PCA) of the LD‐pruned dataset. Colors correspond to population groupings from Figure 1, where blue represents birds from inland sites (allopatric *M. m. melodia*), orange represents coastal mainland sites (parapatric *M. m. atlantica*), and yellow represents southern barrier island sites (allopatric *M. m. atlantica*). The shape of yellow points corresponds to individual barrier islands, where squares are birds from Assateague Island, MD, diamonds are birds from Hog Island, VA, and triangles are birds from Pea Island, NC. *Right*, Genotype composition plot from discriminant analysis of principal components (DAPC) with a priori groupings (labeled below) based on the first 4 principal components (cumulative variance = 9.90%). Bars represent the probability of assignment of each individual to the corresponding clusters. *Top*, individuals split by subspecies (*K* = 2); *center*, individuals split by population grouping (*K* = 3); *bottom*, population groupings with individual barrier islands separated (*K* = 5).

Subsequent PCs were not indicative of population structure, nor significantly informative based on the scree plot (Figure A2, Figure A3). Inland birds showed no substructure in the full‐data PCA, so a separate PCA was conducted on the inland birds alone to determine whether there was any substructure among inland populations. No geographic patterns emerged, consistent with panmixia within the eastern song sparrow across the sampling area (Figure A4). DAPC was able to reliably separate populations at all three levels (subspecies, population, and islands), and the same four birds from the Chesapeake Bay and Delaware exhibited some overlap with the Assateague cluster (Figure 3, Figure A5).

### 3.4 Genome‐Wide Divergence

Genome‐wide *F*_ST_ between the subspecies was low (mean *F*_ST_ = 0.027). Patterns of *F*_ST_ across the genome showed few, isolated regions of the genome with slightly elevated differentiation against the genomic background (maximum window mean *F*_ST_ = 0.430; Figure 4). However, most windows with visually elevated *F*_ST_ had relatively few variant positions, and SNPs in these windows did not individually have exceptionally high *F*_ST_. For example, of the 29 windows with a mean *F*_ST_ > 0.25, there was an average of 17 SNPs per window, versus a genome‐wide average of 363 SNPs per window. Therefore, due to the minimal linkage disequilibrium observed in this system, a window‐based *F*_ST_ outlier approach may lack the resolution needed to reliably identify regions of the genome associated with phenotypic divergence between these subspecies.

**FIGURE 4:**
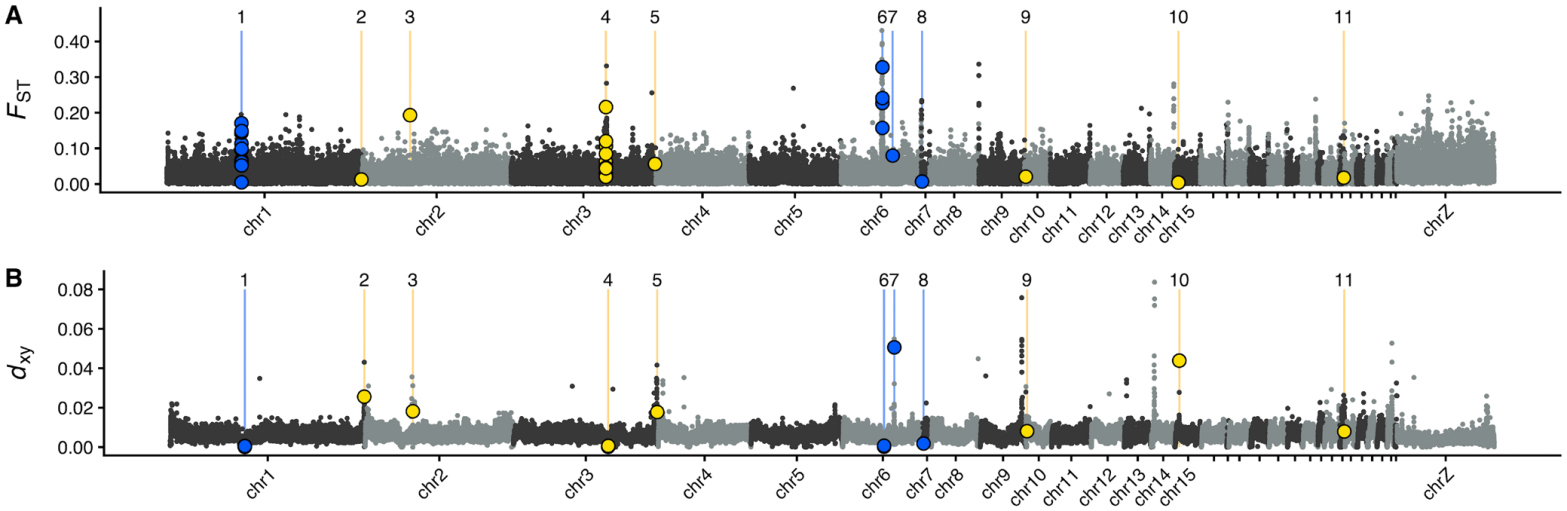
Manhattan plots of genome‐wide statistics of divergence (*F*_ST_ and *d*_xy_) for chromosomal scaffolds in 10‐kbp windows between the barrier islands (*M. m. atlantica*) and inland populations (*M. m. melodia*). Yellow and blue bolded windows contained SNPs identified as under divergent selection from the selection scan (see Figure 6).

Absolute sequence divergence between the subspecies was low (mean *d*_xy_ = 6.00e‐03), tightly correlated with nucleotide diversity for both subspecies (*R* ^2^_*atlantica*_ = 0.983, *R* ^2^_*melodia*_ = 0.988), and incongruent with *F*_*ST*_ (*R* ^2^ = 0.011). The tight correlation between *d*_xy_ and *π*, and their incongruence with *F*_ST_, is consistent with signals of recent divergence and high gene flow observed in other systems (Figure 5; Henderson and Brelsford 2020). Overall, genome‐wide patterns of nucleotide diversity were similar for both subspecies across the genome (*R* ^2^ = 0.951), but average nucleotide diversity was lower in the Atlantic song sparrow (*π*_*atlantica*_ = 5.65e‐03, *π*_*melodia*_ = 6.02e‐03).

**FIGURE 5:**
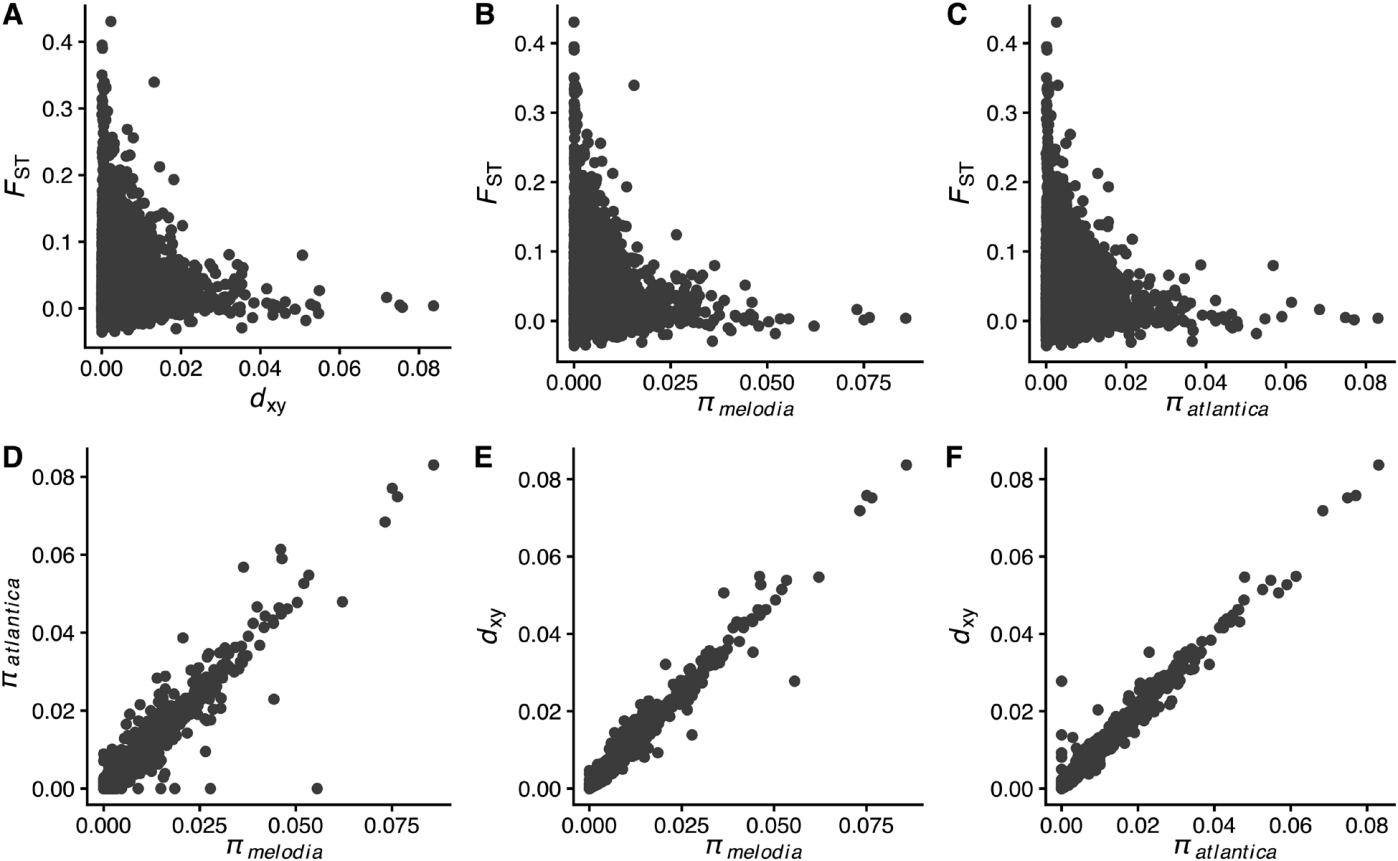
Correlations among *F*_ST_, *d*_xy_, and *π* for the barrier islands (*M. m. atlantica*) and inland (*M. m. melodia*) populations in 10‐kbp windows.

### 3.5 Selection Scan and Candidate Genes

The xpEHH tests for both comparisons yielded several, discrete genome‐wide peaks proximal to genes that exhibited signatures of divergent selection between the subspecies (Figure 6). Several of those peaks (*n* = 11) were shared between both the inland–barrier islands and inland–coastal mainland comparisons (Table 1). Of the gene‐containing genomic regions exhibiting signatures of divergent selection detected in both comparisons, most were under positive selection in the Atlantic song sparrow population (i.e., negative xpEHH; *n* = 7). Three of these genomic regions (i.e., 1, 4, and 6 from Table 1) were flanking putative centromeres, and one of these regions (1 from Table 1) contained a linked set of three genes under selection. Four of these loci were in regions of elevated *F*_ST_ (1, 3, 4, and 6 from Table 1; Figure 4).

**FIGURE 6:**
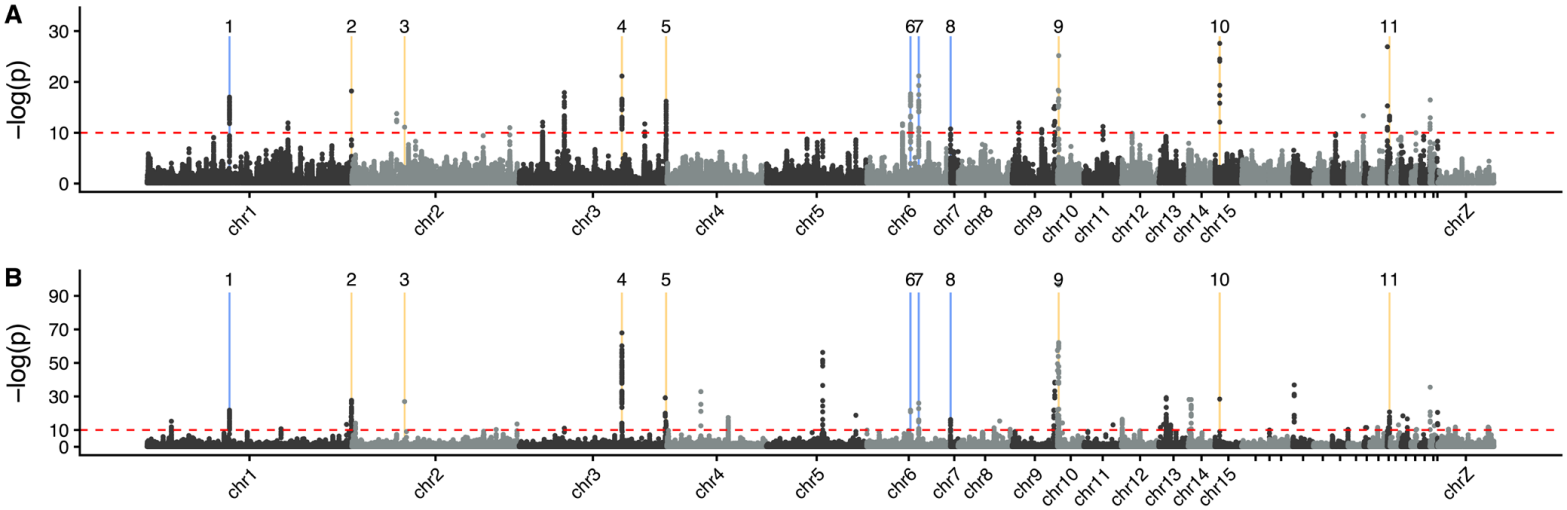
Selection scan results for the (A) inland–barrier islands and (B) inland‐coastal mainland comparisons. Y‐axis shows log‐transformed two‐sided *p*‐values of xpEHH scores with a false discovery rate (FDR) correction. Dashed red line indicates the outlier threshold of ‐log(*p*‐value) > 10. Outlier regions that were detected in both the inland–barrier islands and inland–coastal mainland comparisons are shown with vertical bars in order corresponding to Table 1. Yellow outliers indicate a signature of positive selection in the Atlantic song sparrow population (negative xpEHH), while blue outliers indicate positive selection in the eastern song sparrow population (positive xpEHH).

**TABLE 1:** Genes exhibiting signatures of selection between the Atlantic and eastern song sparrow subspecies based on cross‐population extended haplotype homozygosity (xpEHH) in both the inland–barrier islands and inland–coastal mainland comparisons.

Genomic region	Gene symbol (ID)	Gene name	Chromosome	Hits range	Hits span (bp)	No. Hits	Top hit (xpEHH)
1	*BRD9* (134419636)	Bromodomain‐containing 9	Chr 1 (NC_086194.1, SUPER_1)	79372184–79415327	43,143	21	9.98
	***TRIP13*** (134419650)	Thyroid hormone receptor interactor 13		79398180–79430499	32,319	12	9.13
	***COL15A1*** (134419662)	Collagen type XV alpha 1 chain		79440467–79475189	34,722	15	8.96
2	*ARHGAP39* (134428566)	Rho GTPase activating protein 39	Chr 1 (NC_086194.1, SUPER_1)	172998270	0	1	−10.33
3	*LOC134414286*	C‐type lectin domain family 4 member A‐like	Chr 2 (NC_086195.1, SUPER_2)	65342217	0	1	−8.44
	*LOC134414287*	C‐type lectin domain family 4 member E‐like					
	*LOC134414992*	C‐type lectin domain family 4 member D‐like					
4	***PCSK2*** (134416075)	Proprotein convertase subtilisin/kexin Type 2	Chr 3 (NC_086196.1, SUPER_3)	99952393–100062234	1,09,841	49	−9.66
5	*HADHB* (134416534)	Hydroxyacyl‐CoA dehydrogenase trifunctional multienzyme complex subunit beta	Chr 3 (NC_086196.1, SUPER_3)	139713942–139715185	1243	24	−9.63
	*LOC134416532*	Interferon‐induced very large GTPase 1‐like					
	*LOC134416643*	Sterile alpha motif domain‐containing protein 12‐like					
6	***ANO3*** (134420387)	Anoctamin 3	Chr 6 (NC_086199.1, SUPER_6)	46943224–46971648	28,424	10	9.47
7	*LOC134419818*	Mas‐related G‐protein coupled receptor member H‐like	Chr 6 (NC_086199.1, SUPER_6)	54994563–54994714	151	19	10.86
	*LOC134419819*						
8	*LOC134420903*	Ubiquitin carboxyl‐terminal hydrolase 42‐like	Chr 7 (NC_086200.1, SUPER_7)	25623812–25624848	1036	2	8.13
9	*EFCC1* (134422385)	EF‐hand and coiled‐coil domain containing 1	Chr 10 (NC_086203.1, SUPER_10)	11805592–11806392	800	5	−11.60
10	*LOC134425121*	Semaphorin‐7A‐like	Chr 15 (NC_086208.1, SUPER_15)	3666746–3666806	60	8	−8.41
	***LOC134425495***	Cholesterol side‐chain cleavage enzyme, mitochondrial					
11	*LOC134429203*	Protein S100‐A14‐like	Chr 25 (NC_086218.1, SUPER_25)	11234194–11234225	31	3	−8.50
	*LOC134429347*	Protein S100‐A4‐like					
	***S100A16*** (134429151)	S100 calcium binding protein A16					

*Note:* Statistics presented are from comparison of the barrier islands (*M. m. atlantica*) and inland (*M. m. melodia*) populations. Bolded genes are discussed in the text. Here, hits refers to SNPs with a log‐transformed two‐sided *p*‐value of xpEHH > 10 after a false discovery rate (FDR) correction. Negative xpEHH scores indicate increased haplotype homozygosity (positive selection) in the barrier islands population relative to the inland population, while positive xpEHH scores indicate increased haplotype homozygosity in the inland population.

Several genes were identified in the regions exhibiting signatures of divergent selection between the Atlantic and the eastern song sparrow shared by both the inland–barrier islands and inland–coastal mainland comparisons (*n* = 20; Table 1), some of which had plausible associations with osmoregulatory functions. For example, one gene (*ANO3*) was a member of a chloride transmembrane transporter family (Tian et al. 2012). Other genes had known associations with the kidney, such as *S100A16* and *TRIP13* (Zimmer et al. 2003; Park 2015; Sun et al. 2020, 2022; Jin et al. 2021). A predicted gene (*LOC134425495*) that exhibited signatures of selection was orthologous to *CYP11A1*, a cholesterol side‐chain cleavage enzyme involved in steroidogenesis of hormones such as aldosterone, which thereby plays a role in salt/water balance (Miller and Auchus 2011).

## 4 Discussion

### 4.1 Fine‐Scale Population Structure Within a Specialist Subspecies

Prior efforts were able to discern limited population structure between the Atlantic and eastern song sparrows to some extent, but lacked definitive resolution. Specifically, Clark et al. (2022) delineated the Atlantic and eastern song sparrows as distinct subpopulations using a reduced‐representation sequencing approach, yielding thousands of SNPs but did not clearly differentiate populations nor identify substructure within the Atlantic song sparrow. Ultimately, whole‐genome data and range‐wide sampling were necessary to accurately identify the limited regions of the genome that exhibit signatures of divergent selection between these subspecies and characterize population substructure between and within subspecies.

In the Atlantic song sparrow, we found a complex population structure across its 1000 km breeding range along the Atlantic coast. Coastal mainland sites from the Delaware Bay north to New York constituted one subpopulation, which likely experiences some gene flow from inland populations. The Chesapeake Bay and barrier islands off the coast of Delaware and Maryland made up another distinct subpopulation. Barrier islands off the southern coast of Virginia, which are more isolated from inland birds, made up a third distinct subpopulation. The most southerly and isolated Atlantic song sparrow population, from a barrier island off the coast of North Carolina, represented the most distinct subpopulation. This illustrates that there is likely limited inter‐island gene flow among barrier island populations of the Atlantic song sparrow, and potentially unique standing variation within local island populations.

In contrast to the fine‐scale population structure of the Atlantic song sparrow subspecies, we found panmixia in the eastern song sparrow across a 1500 km north–south sampling area, that is, over 10° of latitude. Populations of the eastern song sparrow from inland sites across the sampled area may have been expected to exhibit isolation by distance (IBD), due to the breadth of its breeding range sampled here. However, panmixia within this subspecies is likely a result of its high dispersal ability and unpredictable migratory behavior (Weatherhead and Boak 1986; Davis and Arcese 1999; Kelly et al. 2016). Whole‐genome studies of some other song sparrow subspecies have not reported population structure within subspecies (Carbeck et al. 2023; Mikles et al. 2023). However, the Channel Islands song sparrow—a nonmigratory subspecies that breeds across several islands off the coast of California—has been shown to exhibit population structure among extant island populations which span a temperature gradient based on reduced‐representation genomic data (Gamboa et al. 2022). Generally, it appears that the extent of substructure varies among song sparrow subspecies due to differences in the extent of geographic isolation, life history traits, and local environmental conditions.

### 4.2 Genomic Architecture of Divergence With Gene Flow

Systems where local adaptation is maintained despite gene flow can shed light on the evolutionary processes that maintain divergence and may ultimately lead to ecological speciation (Rundle and Nosil 2005), particularly the interplay between genomic architecture and selection. We found that loci under divergent selection were generally scattered throughout the genome, rather than clustered in large linkage blocks such as chromosomal inversions. In such a scenario, where multiple unlinked loci contribute to adaptation, maintaining divergence in the face of gene flow likely requires sufficiently strong selection in the absence of assortative mating or other forms of reproductive isolation (Tigano and Friesen 2016).

The selection scan demonstrated the majority of divergent loci were the result of positive selection in the Atlantic song sparrow, consistent with the intense selection pressures of coastal habitats largely driving and maintaining divergence between these subspecies. Notably, a high recombination rate—such as that observed here—can lead to more efficient sorting of co‐adaptive sets of alleles in response to strong selection (Hill and Robertson 1966; Haddrill et al. 2007). We also found that some peaks exhibiting signals of divergent selection based on extended haplotype homozygosity were proximal to putative centromeres, suggesting that the recombination landscape may play a minor role in the maintenance of co‐adapted gene complexes between these subspecies. It is not unprecedented for centromeres to house genes associated with divergence in systems with high gene flow, such as in hybridizing bumble bee species (*Bombus* spp.) where genomic islands of divergence are largely limited to pericentromeric regions throughout the genome (Christmas et al. 2021).

Both temperature and salinity—principal habitat differences between these two subspecies—have been found to be the primary drivers of differentiation among three other coastal song sparrow subspecies in contact that maintain divergence in the San Francisco Bay area (Mikles et al. 2023). Although the locally adaptive phenotypes of the Atlantic song sparrow appear to be somewhat resistant to maladaptive gene flow, demographic modeling suggests that these two subspecies are likely experiencing secondary contact following a period of allopatric divergence, so it is possible this system is not in migration–selection balance and that selection may not be strong enough to prevent the geographically restricted Atlantic song sparrow from being swamped with maladaptive alleles from the eastern song sparrow over time eventually resulting in fusion (Clark et al. 2022).

Notably, *F*_ST_ peaks between the Atlantic and eastern song sparrow subspecies did not consistently correspond with regions exhibiting signatures of divergent selection between the subspecies based on xpEHH. This misalignment between windowed *F*_ST_ and the haplotype‐based selection scan may be a result of extremely low LD—which reduces the power of windowed *F*_*ST*_ to identify regions under divergent selection—incomplete selective sweeps between the populations, and/or gene flow preventing fixation of alleles under divergent selection (Jensen et al. 2016). Many *F*_ST_ peaks represented regions of low genetic diversity in the genome, which can inflate *F*_ST_, rather than regions of elevated divergence driven by selection.

### 4.3 Genetic Basis of Coastal Adaptation

Elucidating the genetic basis of coastal adaptation in the Atlantic song sparrow contributes to our understanding of genotype–phenotype associations in avian traits and provides insights into parallel adaptation to coastal environments in passerines. We identified several genes exhibiting signatures of divergent selection in the Atlantic song sparrow genome, some of which had plausible associations with traits of the avian salt marsh syndrome. For example, several of these genes have known roles in osmoregulatory processes, consistent with the physiological challenges posed by high‐salinity coastal habitats (Greenberg and Maldonado 2006; Greenberg et al. 2006).

Parallel adaptation often arises through lineage‐specific genetic changes. Consistent with the pattern, despite the numerous studies that have examined the genetic basis of coastal adaptation in other passerine species, many genes identified as targets of selection here have not been associated with traits relevant to the salt marsh syndrome in other species previously. For example, none of the genes associated with divergence in the Atlantic song sparrow were identified as candidates of selection in the three coastal song sparrow subspecies of the San Francisco Bay area (Mikles et al. 2023). Similarly, neither a study that characterized the genetic basis of divergent coastal adaptation between the saltmarsh sparrow (*Ammospiza caudacuta*) and Nelson's sparrow ( *A. nelsoni* ; Walsh, Clucas, et al. 2019) nor a study that examined adaptation to hot, saline environments in the mangrove yellow warbler ( *Setophaga petechia* ; Chavarria‐Pizarro et al. 2019) found genes associated with coastal adaptation that were shared with those identified here in the Atlantic song sparrow. Comparisons of freshwater‐, brackish‐, and saline‐adapted subspecies of the Savannah sparrow ( *Passerculus sandwichensis nevadensis* , *P. s*. *alaudinus*, and *P. s*. *beldingi*) in California also found no genes exhibiting signatures of selection in coastal habitats that were shared with those identified here (Benham, Walsh, and Bowie 2024). Notably, two genes identified as putative candidates of selection for the Atlantic song sparrow using reduced‐representation genomic data, *SENP5* and *COL12A1*, were not detected by the selection scan here, and are therefore possibly spurious candidates given the limitation of reduced representation methods to detect genes under selection.

Although some genes identified here are novel candidates of coastal adaptation in a passerine species, some genes under selection in the Atlantic song sparrow have been identified as targets of selections in other coastal–inland passerine subspecies comparisons, emphasizing how parallel evolution to a selective regime is a complex process that can occur through a combination of shared and lineage‐specific genetic mechanisms (Walsh, Benham, et al. 2019). Specifically, two genes, *TRIP13* and *PCSK2*, were divergent between a coastal–inland subspecies pair in a congener, the swamp sparrow ( *M. georgiana georgiana* and *M. g. nigrescens*; Deane‐Coe et al. 2018). *PCSK2* is a convertase with roles cleaving several hormone precursors, including pro‐vasopressin (Pan et al. 2006). *PCSK2* also has been associated with melanin synthesis in avian plumage, as it is the convertase responsible for cleaving ACTH to produce the agonist of MC1R α‐MSH (e.g., Ducrest et al. 2025, Oliver Brown et al. 2025). Another gene detected as a target of selection here, *COL15A1*, has been suggested as influencing variation in body‐size extremes between other song sparrow subspecies, which notably also vary widely in both bill size/shape and plumage pigmentation (Carbeck et al. 2023). Building our understanding of the genetic basis of trait divergence across avian species may help us conserve adaptive variation and understand how population will respond in the face of changing environments.

## Author Contributions

**Jonathan D. Clark:** conceptualization (equal), data curation (lead), formal analysis (lead), investigation (lead), methodology (equal), visualization (lead), writing – original draft (lead), writing – review and editing (equal). **Haw Chuan Lim:** conceptualization (equal), formal analysis (supporting), investigation (supporting), writing – review and editing (supporting). **Brian J. Olsen:** conceptualization (equal), formal analysis (supporting), funding acquisition (supporting), investigation (supporting), supervision (supporting), writing – review and editing (supporting). **Adrienne I. Kovach:** conceptualization (equal), formal analysis (supporting), funding acquisition (lead), investigation (supporting), methodology (equal), supervision (lead), writing – original draft (supporting), writing – review and editing (equal).

## Funding

This work was supported by the Directorate for Biological Sciences (1826777).

## Conflicts of Interest

The authors declare no conflicts of interest.

## Supporting information


**Table S1:** List of samples and metadata.

## Figures and Tables

**FIGURE A1:**
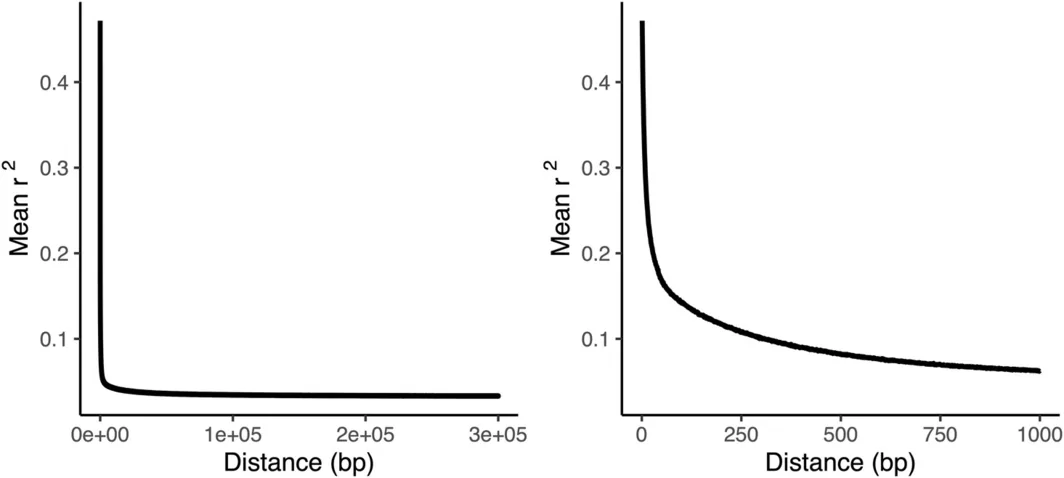
Linkage decay plot. *Left*, Mean linkage (*r* ^*2*^) over a maximum distance of 300 kbp. *Right*, Zoomed in to a maximum distance of 1000 bp.

**FIGURE A2:**
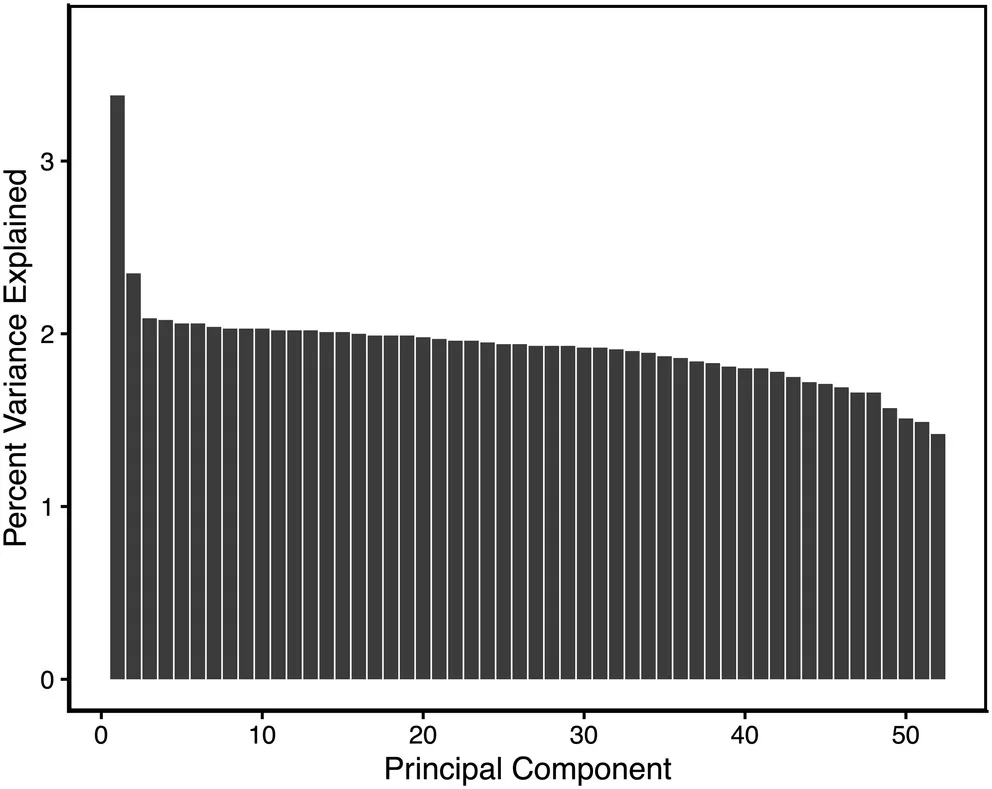
Scree plot of principal components for the full dataset (see Figure 3).

**FIGURE A3:**
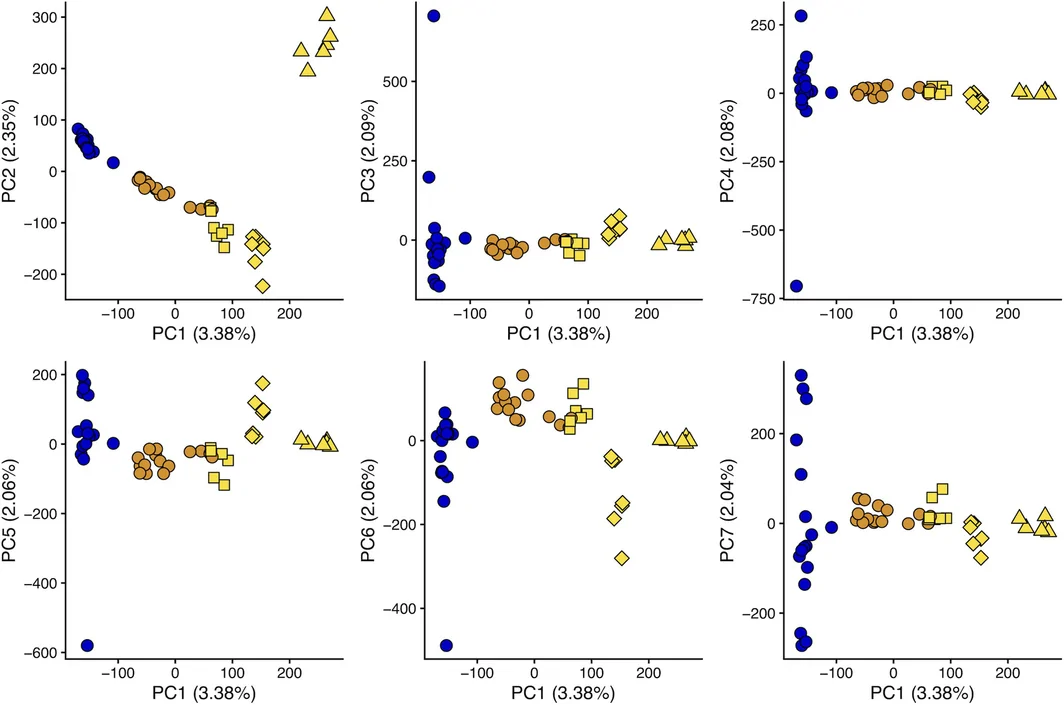
Principal components scatter plots of PC1 through PC7. All *x*‐axes are kept as PC1 for the sake of comparison among plots. Colors correspond to population groupings from Figure 1, where blue represents birds from inland sites (*M. m. melodia*), orange represents coastal mainland sites (*M. m. atlantica*), and yellow represents southern barrier island sites (*M. m. atlantica*). The shape of yellow points corresponds to individual barrier islands, where squares are birds from Assateague Island, MD, diamonds are birds from Hog Island, VA, and triangles are birds from Pea Island, NC. Though PC3–PC7 represent variation among inland birds, they are not generally geographically informative (see Figure A4).

**FIGURE A4:**
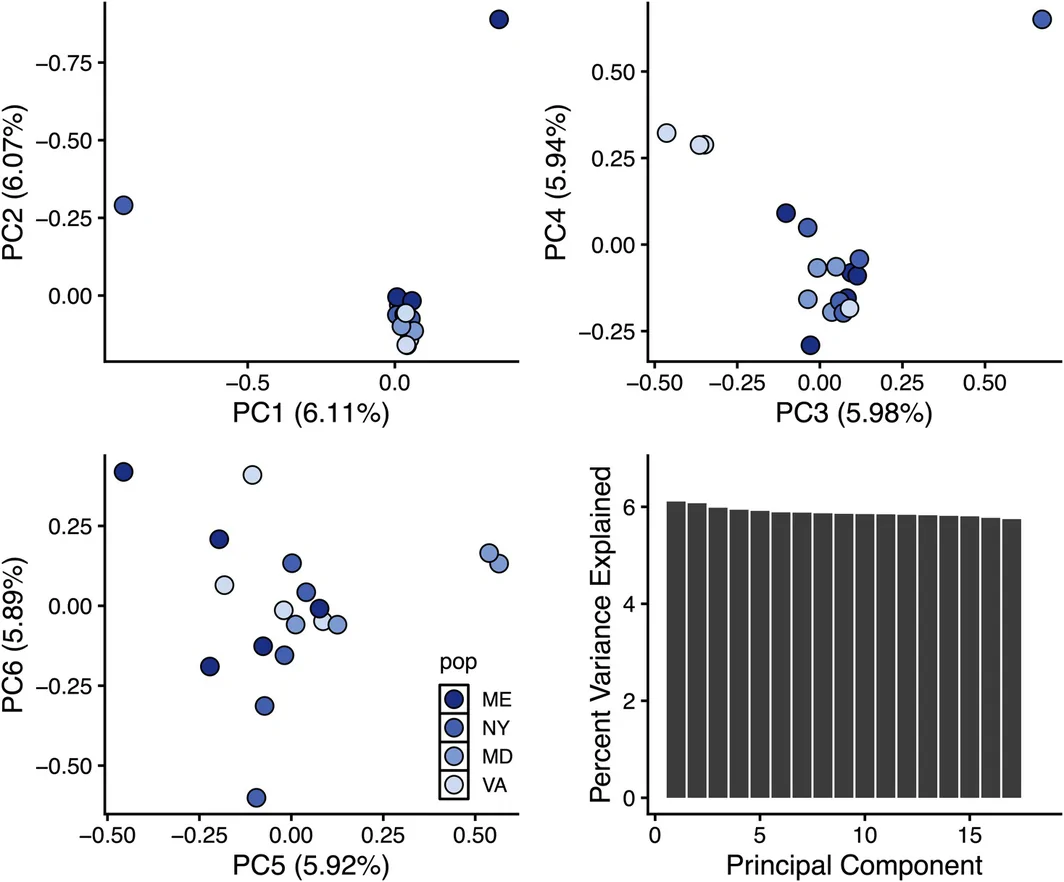
Principal components analysis of only inland birds. No clear geographic patterns emerge, and the scree plot shows uniform variance explained across PCs, consistent with panmixia across the sampled area.

**FIGURE A5:**
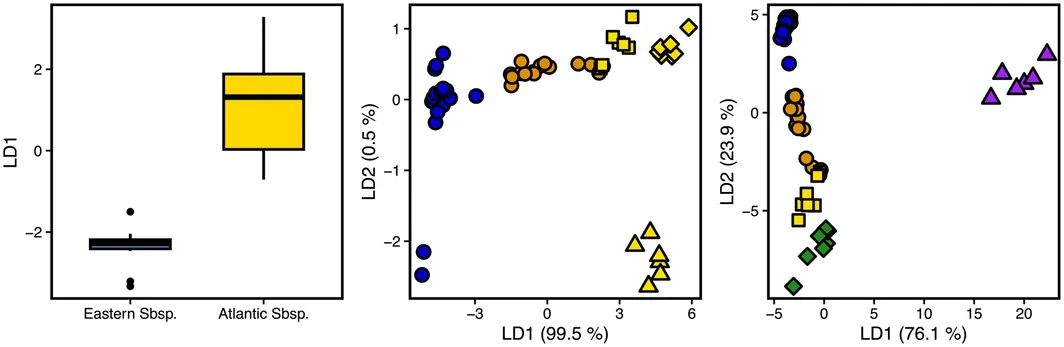
DAPC plots with a priori groupings at varying levels of *K* with the first 4 principal components retained. *Left*, Birds grouped by subspecies (*K* = 2). *Center*, Birds separated by population grouping (*K* = 3). *Right*, Each barrier island split out (*K* = 5). All colors correspond to a priori group assignment as in Figure 3 and point shapes correspond to sampling location as in Figure 2.

## Data Availability

Raw sequencing reads are available on the NCBI Sequence Read Archive (SRA) under accession code PRJNA1138247. VCFs of individual genotype data and outputs of the selection scan are available on Dryad (DOI: 10.5061/dryad.1c59zw44n).
